# Supplementary material for: Sustained Improvement in the Management of Patients with Non-Small-Cell Lung Cancer (NSCLC) Harboring ALK Translocation: Where Are We Running?
Source: Curr Oncol. 2023 May 16;30(5):5072–92. doi: 10.3390/curroncol30050384 (PMC10217667; doi:10.3390/curroncol30050384)
Supplement: Supplementary file 1 [file curroncol-30-00384-s001.zip › curroncol-2327625-supplementary.pdf]

Table S1 Main features of the first-line clinical trials of ALK-Is

**Trial**	**Ref.**	**Detection of ALK**	**Lab**	**Stratification factors**
PROFILE-1014	Solomon 2014	FISH	Central lab	ECOG PS; BM; Ethnicity
ASCEND-4	Soria 2017	IHC	Central lab	ECOG PS; BM; prior CT
ALEX	Peters 2017	IHC	Central lab	ECOG PS; BM; Ethnicity
ALTA-1L	Camidge 2018	FISH/IHC	Local lab	BM; prior CT
EXALT-3	Horn 2021	FISH/IHC	Local lab*	ECOG PS; BM; Ethnicity; prior CT
The Crown	Shaw 2020	FISH/IHC	Local lab	BM; Ethnicity

*after protocol amendment all cases have been detected by a central lab; abbreviations: ref.= reference; FISH = Fluorescence In Situ Hybridization; IHC= immunohistochemistry; Lab= laboratory; ECOG PS= Eastern Cooperative Oncology Group performance status; BM = brain metastases

Table S2 patients with baseline brain metastases according the several randomized trials

Trial [drug]	Pts with BM	Prior RT	% of BM+	% all pts
PROFILE-1014[crizotinib]	92	92	100	27
ASCEND-4 [ceritinib]	121	50	41	13
ALEX [alectinib]	122	46	38	15
ALTA-1L [brigatinib]	96	37	39	13.5
eXalt3 [ensartinib]	104	14	13.5	5
The Crown [lorlatinib]	78	19	24	6

Abbreviations: Pts = patients; BM = brain metastases; RT = radiotherapy.

Solomon BJ JCO 2016(Profile 1014 all treated BM ic-DCR at 24 wks); Soria JC Lancet 2017(Ascend-4) 50 pts RT; Camidge DR JTO 2019 (Alex) 46 RT (15%); Popat S ESMO 2021 (ALTA-1L) 37 (13%) rt; Horn L Jama Onc 2021 (eXalt3) 14 PTS (5%) RT; Shaw AT NEJM 2020 (Crown) 19 RT (6%)

Table S3 Bypass signaling pathways associated with resistance to ALK inhibitors

**Reference**	**Drug**	**Molecular Pathway**	**Setting**
Sasaki 2011[88]	Crizotinib	ALK L1152R + EGFR coactivation	DFCI076 cell line + 3/50 pts (6%)
Miyawaki 2017[89]	Ceritinib/alectinib	EGFR coactivation	H3122-CER cell lines
Katayama 2012[71]	Crizotinib	Kit amplification + EGFR coactivation	18 pts: 2/18 pts KIT; 9/18 EGFR
Lowly 2014[90]	Crizotinib	IGF1R coactivation	H3122 cell lines
Crystal 2014[91]	Crizotinib	MAP2K1 mutation	Cell line derived from a pt
Tsuji 2019[92]	Alectinib	c-Src and MET coactivation	Two pt-derived cell lines
GoujI 2014[93]	Alectinib	MET amplification	Case report
Shi 2020[94]	Alectinib	BRAFV600E + MET amplification	pt-derived xenograft models
Yun 2019[95]	ALK-Is	YAP oncogenes induction	pt-derived xenograft models
Yang 2021[81]	Crizotinib/ensartinib	P53 and several pathways	Phase II trial of ensartinib
Recondo 2020[83]	lorlatinib	ALK compound mutations + NF2 loss function mutations	5 pt-derived xenograft models

Abbreviations: EGFR Epidermal Growth Factor Receptor; pt(s) = patient(s); KIT = tyrosine-protein kinase; IGF1R = Insulin Growth Factor 1 Receprtor; MAP2K1 = Mitogen-activated protein 2 kinase 1; c-Src = proto-

oncogene Src; MET = met proto-oncogene (MET) gene; BRAF = proto-oncogene B-Raf; YAP= Yes Associated Transcriptional Regulator; superscripted numbers indicate references cited in the text.

Table S4 Ongoing trials of blood-assessed NGS to detect ALK+ disease

Trial	Phase	Pt N	Drug	Class of drug	Setting	Aim	Trial Identifier
LIQUIK	Obs.	200	NA	NA	Naïve	Non-inferiority of LB	NCT04703153
LIBIL	Obs.	900	NA	NA	Naive	Identification of the genetic profile	NCT02511288
BFAST	II/III	1000	Alectinib	2nd ALK-I	Naïve ALK-I	Activity of alectinib on blood detected ALK	NCT03178552
	II	35	Brigatinib	2nd ALK-I	Refractory ALK-I	Activity of brigatinib on blood detected ALK	NCT04074993

Abbreviations: NGS = next generation sequencing; Pt N= patient number; Obs. = observational; ALK-I = ALK inhibitor(s); LB = liquid biopsy

Table S5 summary of ongoing clinical trials of ALK-Is in the early stage disease

Trial	Phase	Pt number (stage)	Drug(s)	Principal endpoint
Neoadjuvant				
NAUTIKA1 (NCT04302025)	II	80 pts (IB-IIIB, 8th TNM)	Alectinib x 2 cycles	MPR
ALNEO	II	33 pts (III 8th TNM)	Alectinib x 2 cycles	MPR
NCT05380024	II	10 pts (IIA-IIIB 8th TNM)	Ensartinib x2 cycles	MPR
NCT05361564	II	12 pts (I-IIIA 8th TNM)	Brigatinib From 4 to 10 weeks	Drug resistance
NCT04197076	Observational	200 pts (IIB-IIIB 8th TNM)	ALK-Is X 2-3 cycles	DFS/pCR
Adjuvant				
ALCHEMIST-EA4512 (NCT02201992)	RPIII	168 pts (IB-IIIA 7th TNM)	Crizotinib X 2 years Vs observation	OS
ALINA (NCT03456076)	RPIII	255 pts (IB-IIIA 7th TNM)	Alectinib x 2 years Vs platinum-based CTx 4 cycles	DFS
NCT05186506	RPII	152 pts (II-IIIA 7th TNM)	Ensartinib x 2 years Vs platinum-based CT x 4 cycles	DFS
NCT05341583	RPIII	202 pts (II-IIIB 8th TNM)	Ensartinib x 2 years Vs placebo	DFS
Unresectable III stage after CRT				
NCT05170204	I-RPIII	320 pts Post CRT	Alectinib x 3 yrs Vs durvalumab x 1 year	PFS
BOUNCE (NCT05718297)	RPII	44 pts after CRT	Brigatinib x 3 years Vs observation or durvalumab	PFS
NCT05351320	Ib	40 pts concomitant CRT	WX-0593 x 2 cycles plus CRT -> WX-0593 up to PD	% G>3 pneumonitis

Abbreviations: Pt(s) = patient(s); RP = randomized phase; CRT = chemoradiotherapy; MPR = major pathological response; pCR = pathological complete response; OS = overall survival; DFS = disease-free survival; PFS = progression-free survival; G = grade
